# Risk factors for carpal tunnel syndrome or trigger finger following distal radius fracture: a nationwide study

**DOI:** 10.1038/s41598-020-57415-x

**Published:** 2020-01-16

**Authors:** Kuang-Ting Yeh, Ru-Ping Lee, Tzai-Chiu Yu, Jen-Hung Wang, Kuan-Lin Liu, Cheng-Huan Peng, Hao-Wen Chen, Ing-Ho Chen, Chung-Yi Hsu, Cheng-Li Lin, Wen-Tien Wu

**Affiliations:** 1 Department of Orthopedics, Hualien Tzu Chi Hospital, Buddhist Tzu Chi Medical Foundation, Hualien, Taiwan; 2 School of Medicine, Tzu Chi University, Hualien, Taiwan; 3 Institute of Medical Sciences, Tzu Chi University, Hualien, Taiwan; 4 Department of Research, Hualien Tzu Chi Hospital, Buddhist Tzu Chi Medical Foundation, Hualien, Taiwan; 5 Graduate Institute of Clinical Medical Science, China Medical University, Taichung, Taiwan; 6 Management Office for Health Data, China Medical University Hospital, Taichung, Taiwan

**Keywords:** Trauma, Epidemiology, Risk factors, Comorbidities, Disability

## Abstract

New-onset carpal tunnel syndrome (CTS) and trigger finger after distal radius fractures (DRFs) with or without open reduction and internal fixation (ORIF) have been reported inconsistently across different studies. This study assessed the incidence of CTS and trigger finger after DRFs using Taiwan National Health Insurance Research Database. In total, 1454 patients in the case (ORIF) cohort and 1454 patients in the control (non-ORIF) cohort were included in this retrospective study. The mean age was approximately 55 years old, and the female to male ratio was approximately 3/2. Nine patients underwent carpal tunnel release (CTR) surgery after diagnosis of CTS in the case group, and no patients did in the control group; whereas 19 cases of CTS were diagnosed without CTR in the case group, and 4 such cases were observed in the control group. Five cases of trigger finger were diagnosed in the case group, and 3 cases were diagnosed in the control group. CTS were significantly associated with ORIF for DRFs within 9 months after the fracture, whereas trigger finger was not significantly different between groups. Diabetes mellitus was a significant risk factor for CTS and trigger finger within 9 months after the incidence of DRFs.

## Introduction

Distal radius fractures (DRFs) are the most common fractures seen in the emergency department and account for approximately 25% of fractures in the paediatric population and up to 18% of all fractures in the elderly age group; however, they have a considerable effect on the life quality of younger adults^1^. Open reduction and internal fixation (ORIF) with volar plating for DRFs is a standard choice for treatment, but the common complications include arthrosis, tendon ruptures, complex regional pain syndrome, wrist or finger stiffness, and carpal tunnel syndrome (CTS)^2^. Flexor tendon injuries after ORIF for DRFs may further cause trigger finger, but reports regarding this complication are rare^3,4^. The carpal tunnel is the passageway deep to the transverse carpal ligament between the tubercles of the scaphoid and trapezoid bones on the radial side and the pisiform and hook of the hamate on the ulnar side. The pressure in the carpal tunnel can easily be increased by a space-occupying lesion or the inflammatory condition after DRFs, which results in median nerve compression, a measurement pathognomy for CTS^5^. CTS after DRFs can be classified into 3 types by the onset and development of symptoms: acute, transient, or delayed. Acute CTS may often require emergent release surgery, and observation is initially sufficient for the other two types^6^. Trigger finger often results from the mismatch of the flexor tendons and the surrounding retinaculum of the A1 pulley, and it may be caused by postoperative wrist or finger stiffness after a DRF. Epidemiology studies have suggested that CTS can occur concomitantly with trigger finger^7,8^. This study analysed nationwide population-based retrospective cohort data to assess the incidence of complications, especially trigger finger and CTS, after ORIF for DRFs.

## Results

**Figure 1:**
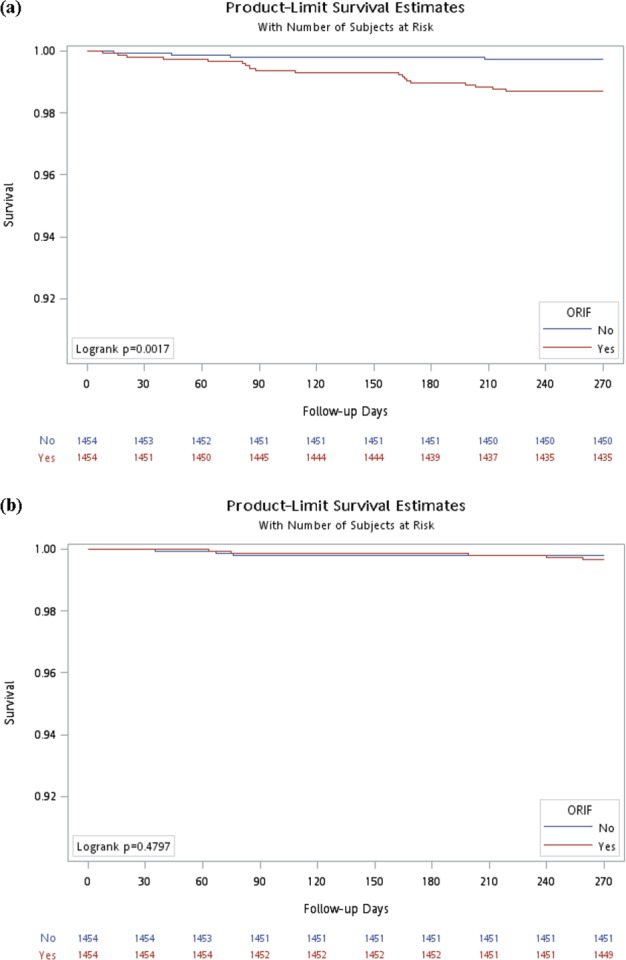
Cumulative incidences of CTS (**a**) and trigger finger (**b**) in both case (ORIF) and control (non-ORIF) groups using the Kaplan–Meier method. The cumulative incidences of CTS in patients in the case and control groups differed significantly, whereas those of trigger finger in both groups exhibited no significant difference.

We included 1454 patients in the case cohort and 1454 patients in the control cohort. The age and sex distributions of the 2 cohorts were similar. The mean age was approximately 55 years old, and the female to male ratio was approximately 3/2 (Table 1). More than 90% of the patients had DRFs from 2004 to 2012. The incidences of the comorbidities did not significantly differ between the 2 cohorts. Approximately 40% of the patients had hypertension (HTN), 30% had hyperlipidaemia, 20% had diabetes mellitus (DM), 20% had osteoporosis, 10% had depression, and 2% had chronic renal failure (Table 1). There were 32 patients who were diagnosed as CTS and the other 8 patients diagnosed as trigger finger in this study. There was no patient who had both CTS and trigger finger. We divided the case of CTS into those who had received CTR and those who had not received CTR as two different degree of tolerability of the symptoms, which an indicator of severity. Nine patients underwent carpal tunnel release (CTR) surgery after a diagnosis of CTS in the case group, and no patients underwent CTR in the control group; whereas CTS was diagnosed without CTR in 19 patients in the case group and 4 cases in the control group, with an adjusted hazard ratio (aHR) of 4.76 (95% confidence interval [CI] = 1.62–14, *p* < 0.01) (Table 2). There were 5 cases where trigger finger was diagnosed in the case group and 3 cases in the control group. The presence of DM significantly increased the incidences of CTS (aHR of 2.76; 95% CI = 1.04–6.36, *p* < 0.05) and trigger finger (aHR of 6.53; 95% CI = 1.56–27.34, *p* < 0.05) after DRFs. The cumulative incidences of CTS increased in the case groups significantly than those in control group (log-rank test: *p* < 0.001), whereas those of trigger finger in both groups exhibited no significant difference (Fig. 1).

**Table 1:** Demographic characteristics of the DRFs patients with and without ORIF in Taiwan. DRFs: distal radius fracture. ORIF: open reduction internal fixation. SD: standard deviation.

Variable	ORIF				p
	No (Control cohort) n = 1454		Yes (Case Cohort) n = 1454		
	n	%	n	%	
**Sex**					0.9395
Female	882	60.66	884	60.8	
Male	572	39.34	570	39.2	
**Age at baseline, years**					0.0649
<40 years	320	22.01	295	20.29	
40-64 years	668	45.94	731	50.28	
≥65 years	466	32.05	428	29.44	
Mean (SD)	55.08 (17.91)		54.79 (16.73)		0.6544
**Index year**					0.2410
2000-2003	98	6.74	121	8.32	
2004-2007	682	46.91	658	45.25	
2008-2012	674	46.35	675	46.42	
**Comorbidities**					
Hypertension	577	39.68	594	40.85	0.5204
Diabetes mellitus	294	20.22	296	20.36	0.9265
Hyperlipidemia	416	28.61	451	31.02	0.1559
Osteoporosis	280	19.26	314	21.6	0.1178
Chronic renal failure	27	1.86	32	2.2	0.5108
Depression	129	8.87	150	10.32	0.1861

**Table 2:** Cox model with hazard ratios and 95% confidence intervals of carpal tunnel syndrome and trigger finger associated the diagnosis of DRFs in the study population.

Variable	CTS undergo CTR			CTS not undergo CTR			Trigger finger		
	no. (n = 9)	Crude HR (95% CI)	Adjusted HR (95% CI)^‡^	no. (n = 23)	Crude HR (95% CI)	Adjusted HR (95% CI)^‡^	no. (n = 8)	Crude HR (95% CI)	Adjusted HR (95% CI)^‡^
**ORIF**									
No	0	1 (reference)		4	1 (reference)	1 (reference)	3	1 (reference)	1 (reference)
Yes	9			19	4.77 (1.62,14.02)**	4.76 (1.62,14)**	5	1.67 (0.4,6.97)	1.61 (0.38,6.78)
**Sex**									
Female	6	1 (reference)		15	1 (reference)	1 (reference)	8		
Male	3	0.77 (0.19,3.09)		8	0.83 (0.35,1.95)	0.96 (0.38,2.42)	0		
**Age at baseline, years**									
<40 years	1	1 (reference)		4	1 (reference)	1 (reference)	0		
40-64 years	4	1.76 (0.2,15.75)		11	1.21 (0.38,3.79)	0.92 (0.28,3.05)	7		
≥65 years	4	2.76 (0.31,24.66)		8	1.37 (0.41,4.56)	0.9 (0.23,3.47)	1		
**Index year**									
2000-2003	0	1 (reference)		0			1	1 (reference)	1 (reference)
2004-2007	4			10			3	0.49 (0.05,4.71)	0.51 (0.05,4.95)
2008-2012	5			13			4	0.65 (0.07,5.82)	0.65 (0.07,5.84)
**Comorbidities** (**Yes vs. No**)									
Hypertension	5	1.86 (0.5,6.91)		14	2.31 (1,5.33)		4	1.48 (0.37,5.94)	
Diabetes mellitus	3	1.97 (0.49,7.87)		9	2.53 (1.1,5.86)*	2.57 (1.04,6.36)*	5	6.56 (1.57,27.46)*	6.53 (1.56,27.34)*
Hyperlipidemia	3	1.18 (0.29,4.71)		10	1.81 (0.79,4.13)		5	3.93 (0.94,16.44)	
Osteoporosis	3	1.95 (0.49,7.8)		5	1.08 (0.4,2.91)		2	1.3 (0.26,6.44)	
Chronic renal failure	0			0			0		
Depression	2	2.7 (0.56,12.99)		5	2.64 (0.98,7.11)		2	3.15 (0.64,15.61)	

CTS: carpal tunnel syndrome. HR: Hazard Ratio; ORIF: open reduction internal fixation. ^‡^Adjusted hazard ratio: Multivariate Cox regression model adjusted for gender, age, index year, and significant variables in crude Cox regression model.

*p < 0.05, **p < 0.01, ***p < 0.001.

## Discussion

The results of our study revealed a significant correlation between DRFs with ORIF (case cohort) and the incidence of CTS at months posttrauma. Most patients did not need CTR, but those who required CTR were all in the case cohort. Previous studies have speculated as to the causes of median nerve injury or compression after DRF^9–11^. A retrospective study of the proposed risk factors for acute CTS in 50 cases of DRFs concluded that ipsilateral upper extremity trauma proximal to metacarpal bones and translation of the fracture fragments could be significant predictors of acute CTS^12^. A retrospective review of 96 DRFs treated with volar locked plate fixation revealed that 15 of the patients had median nerve dysfunction and 2 of them required further CTR surgery. The rate of complications appeared to decrease with surgeon experience^13^. Odulama *et al*. studied 69 patients with displaced DRFs treated with volar plate fixation and reported 17 cases of median neuropathy, with 8 of these patients having received prophylactic CTR surgery. Six of the 9 cases that had not received prophylactic CTR surgery were resolved spontaneously, and the other 3 required CTR^14^. Furthermore, ORIF for DRFs may cause a bowstring effect between the divisions of the flexor retinaculum and an increase of the friction force of the flexor tendons on the A1 pulleys, further manifesting newly onset trigger finger^15^. Poor rehabilitation after the operation, which results in wrist and finger stiffness, may cause triggering problems of the flexor tendons. In Fig. 1 the results revealed that the cumulative incidence increased in both groups of DRFs and it increased more significantly in the group of ORIF for DRFs than the other group. The results pointed that CTS would occur after DRFs, but ORIF was an important factor increasing the incidence of CTS. These complications may be caused by further soft tissue damage caused by surgery, poor rehabilitation after the surgery, and the tenting of the implant placed in the fracture site. From the findings of our result and Odulama’s study^14^ we can conclude that CTS may be an important complication of ORIF for DRF, but prophylactic CTR seems to be not beneficial for these patients. We should focus on the less traction during operation and acceleration method of soft tissue recovery.

In this study, we observed no significant relationship between ORIF and new-onset trigger finger; however, the patient number was slightly higher in the case cohort. The reason we chose 9 months as the interval after trauma in this study was that the incidence of the complications could be more related to DRFs during the recovery period. Further investigation of the onset of trigger finger after DRFs with larger case numbers and a longer period of follow-up can be performed in the future.

In the study, patients with DRFs complicated with CTS and trigger finger during the 9 months following trauma had a significantly higher percentage of DM compared with those patients who did not experience these complications. Because DM often is accompanied by peripheral neuropathy and peripheral swelling, CTS and trigger finger can be common complications. A systemic review and meta-analysis conducted by Pourmemari and Shiri based on 25 studies with a total of 92,564 cases revealed that both Type 1 and Type 2 DM are risk factors for CTS^16^. Another study concluded that nerve conduction measurements of the median nerve tended to differ between patients with and without DM^17^. Another study from the Taiwan National Health Insurance Research Database (NHIRD) revealed that the prevalence rates of 41,871 Taiwanese were 1.59% for CTS and 1.07% for trigger finger; the related risks included being female and between 50 and 59 years old, comorbidity with rheumatoid arthritis or diabetes, and use of hormone antagonists^18^. Further detailed research on the relationship between the peripheral neuropathy, tendinopathy, and severity of DM can be investigated in the future.

The advantage of this study is that a large sample was used to analyse trigger finger and CTS risk differences between DRF patients who did or did not receive ORIF. Moreover, selection and nonresponse biases may have been minimised by the comprehensive coverage of the National Health Insurance system (>96% of the population). However, the study has some limitations. First, we could not balance the severity of CTS and trigger finger between the 2 groups because the Longitudinal Health Insurance Database 2000 (LHID2000) does not include information regarding symptoms and electrophysiological findings. Second, although we divided the outcomes of CTS into those who did and did not require CTR, the number of complicated cases was insufficient to demonstrate significance, as were the outcomes of trigger finger. Third, the degree and classification of fracture may greatly influence of the soft tissue of the wrist and the incidence of CTS or trigger finger. However, in the research database the relationship between X-ray parameters of DRFs and the related complications were not available for analysis. More cases and a longer study period may be required for stronger evidence of the correlation between the 2 complications and DRFs.

In conclusion, from this study CTS were significantly associated with ORIF for DRFs within 9 months after the fracture, whereas trigger finger was not significantly different between groups. Diabetes mellitus was a significant risk factor for CTS and trigger finger within 9 months after the incidence of DRFs. The surgery related factors should be considered for preventing the complications, such as decreasing intraoperative soft tissue dissection, retraction and accelerating soft tissue healing ability with early mobilization, especially for the patients with diabetes. The prophylactic CTR for ORIF of DRF may not be necessary due to low incidence in our study and not beneficial according to the literature review.

## Materials and Methods

### Data source

The LHID2000, a subset of the NHIRD, includes almost the entire population of Taiwan. Started in 1995, the National Health Insurance programme provides all-round medical care, including outpatient and inpatient care, for nearly 99% of the 23.74 million citizens of Taiwan^19^. The LHID2000 contains the data of 1 million beneficiaries randomly selected from the 2000 registry for beneficiaries of the NHIRD. These random samples (LHID2000) have been confirmed by the National Health Insurance to be representative of residents of Taiwan. For each beneficiary, a unique identification number is used to link all insurance information and healthcare records. Diseases are defined by the International Classification of Diseases, 9th Revision, Clinical Modification (ICD-9-CM) codes. The Research Ethics Committee of China Medical University and Hospital in Taiwan approved the research (CMUH-104-REC2-115). All the research methods performed in this study have been confirmed as accordance with the relevant guidelines and regulations. Because LHID2000 was a legal and delinked database for research, this study is exempt from the informed consents of people by law.

### Sample design

The cohort study targeted NHIRD insurants of age ≥20 years as new incident cases of DRFs (ICD-9-CM codes: 813.40–42, 813.44–45, 813.47, 813.50–52, 813.54) between 2000 and 2012. The case and control cohorts were respectively the patients who received ORIF (ICD-9-CM procedure: 79.32) and the patients who received methods other than ORIF. The outcomes included CTS (ICD-9-CM code: 354.0) and trigger finger (ICD-9-CM code: 727.03). The index date for both cohorts was the date of the incidence of DRF. Any patient diagnosed with trigger finger or CTS 9 months before the index dates, having multiple injuries (ICD-9-CM 959.99), or missing data of sex or age was eliminated. The follow-up period was at most 12 months. Based on a logistic regression model, we propensity-score-matched age, sex, and index date in a 1:1 manner. We evaluated the distributions of case and control cohorts by sex, age, index year, and comorbidity including HTN (ICD-9-CM code 401–405), DM (ICD-9-CM code 250), hyperlipidaemia (ICD-9-CM code 272), osteoporosis (ICD-9-CM codes 733.00–09), chronic renal failure (ICD-9-CM codes 585.1.00–585.9), and depression (ICD-9-CM codes 296.2, 296.3, 296.82, 300.4, 309.0, 309.1, 311).

### Statistical analysis

We applied standardised mean difference on strata of sex, age, comorbidity, means of age, and follow-up period. The incidence rate was defined as the number of events divided by person–years. We obtained the crude hazard ratios, aHRs, and 95% CIs based on a multivariable Cox proportional hazard regression model for sex, age, and comorbidity. We used the Kaplan–Meier method to derive the cumulative incidences of CTS and trigger finger among patients with DRFs, and we performed the log-rank test to examine their significance. The analyses and data were performed in SAS 9.4 software (SAS Institute, Cary, NC, USA). Significance was considered *p* < 0.05.

## Conclusion

CTS was significantly associated with ORIF for DRFs within 9 months after the fracture, whereas trigger finger was not significantly different between groups. DM is a significant risk factor of CTS and trigger finger within 9 months of DRFs.

## Data Availability

All data generated or analysed during this study are included in this article and its supplementary information files.
